# Exercise prevents impaired autophagy and proteostasis in a model of neurogenic myopathy

**DOI:** 10.1038/s41598-018-30365-1

**Published:** 2018-08-07

**Authors:** Juliane C. Campos, Leslie M. Baehr, Kátia M. S. Gomes, Luiz R. G. Bechara, Vanessa A. Voltarelli, Luiz H. M. Bozi, Márcio A. C. Ribeiro, Nikolas D. Ferreira, José B. N. Moreira, Patricia C. Brum, Sue C. Bodine, Julio C. B. Ferreira

**Affiliations:** 1 Institute of Biomedical Sciences, University of Sao Paulo, Sao Paulo, 05508-000 Brazil; 2 Department of Internal Medicine, Endocrinology and Metabolism Division, University of Iowa, Iowa, 52242 USA; 3 School of Physical Education and Sport, University of Sao Paulo, Sao Paulo, 05508-030 Brazil; 4 Cardiac Exercise Research Group, Faculty of Medicine and Health Sciences, Norwegian University of Science and Technology, Trondheim, 7006 Norway

## Abstract

Increased proteolytic activity has been widely associated with skeletal muscle atrophy. However, elevated proteolysis is also critical for the maintenance of cellular homeostasis by disposing cytotoxic proteins and non-functioning organelles. We recently demonstrated that exercise activates autophagy and re-establishes proteostasis in cardiac diseases. Here, we characterized the impact of exercise on skeletal muscle autophagy and proteostasis in a model of neurogenic myopathy induced by sciatic nerve constriction in rats. Neurogenic myopathy, characterized by progressive atrophy and impaired contractility, was paralleled by accumulation of autophagy-related markers and loss of acute responsiveness to both colchicine and chloroquine. These changes were correlated with elevated levels of damaged proteins, chaperones and pro-apoptotic markers compared to control animals. Sustained autophagy inhibition using chloroquine in rats (50 mg.kg^−1^.day^−1^) or muscle-specific deletion of Atg7 in mice was sufficient to impair muscle contractility in control but not in neurogenic myopathy, suggesting that dysfunctional autophagy is critical in skeletal muscle pathophysiology. Finally, 4 weeks of aerobic exercise training (moderate treadmill running, 5x/week, 1 h/day) prior to neurogenic myopathy improved skeletal muscle autophagic flux and proteostasis. These changes were followed by spared muscle mass and better contractility properties. Taken together, our findings suggest the potential value of exercise in maintaining skeletal muscle proteostasis and slowing down the progression of neurogenic myopathy.

## Introduction

Skeletal muscle atrophy and dysfunction are hallmarks of several degenerative processes^1^. Considering the continuous increase in lifespan, it is expected that pathology- or disuse-induced muscle weakness/wasting will likely affect every person during lifetime^2^. Therefore, a better understanding of the cellular and molecular signaling pathways involved in skeletal muscle pathophysiology as well as the development of pharmacological and non-pharmacological interventions are critical to improve quality of life in the long-term.

Skeletal muscle is an extremely plastic tissue that modifies its size through adjustments in both protein synthesis and degradation. Increased proteolytic pathways (i.e. autophagy and ubiquitin-proteasome system) have been extensively associated with loss of muscle mass^3,4^. However, proteolysis is also crucial for the maintenance of proteostasis and cellular homeostasis^5^. We and others have previously reported that proteostasis disruption contributes to the onset and progression of many degenerative diseases through the accumulation of damaged proteins (i.e. misfolded proteins)^6,7^. Therefore, a better understanding of proteolytic pathways involved in tissue proteostasis during both physiological and pathological conditions is critical for developing better therapies against degenerative diseases such as skeletal myopathies.

Autophagy (also referred as macroautophagy) plays a critical role in protecting post-mitotic cells, including skeletal muscle, from stress–induced toxicity^8^. This highly conserved proteolytic pathway – driven by more than 30 components, coordinates and oversees the clearance of damaged proteins and organelles by engulfing cytosolic material into autophagosomes (double-membraned vesicles) and assisting them to fuse with lysosomes for degradation^9,10^. Autophagy-related markers are usually elevated in different models of skeletal muscle atrophy/dysfunction^11–13^ and impaired autophagy is associated with severe skeletal myopathy-related diseases such as Pompe, Danon and MDC1A^5^. Moreover, the inability to induce autophagy under stress results in a more pronounced skeletal myopathy in both rodents and humans^14–17^.

Skeletal muscle-related properties (i.e. grip strength, neuromuscular tasks, aerobic exercise capacity) are considered independent predictors of survival, and even small increases in general muscle function reflects into a better quality of life and lower risks of mortality^18,19^. Therefore, there is a clinical need in preventing or delaying the onset of skeletal muscle degeneration in order to extend healthspan. In this regard, exercise has been used as a safety non-pharmacological strategy capable of attenuating muscle weakness/wasting in both health and disease. We recently reported that exercise protects failing hearts by activating autophagy and maintaining proteostasis^20–22^. Moreover, autophagy seems to be critical to induce skeletal muscle remodeling triggered by exercise^23^. Despite the prominent role of autophagy and protein homeostasis in maintaining muscle physiology, the impact of exercise on protein quality control in skeletal myopathies is not fully understood. Here, we set out to determine the role of exercise in preventing the impairment of skeletal muscle autophagy and proteostasis as well as its impact on muscle mass and contractility properties in a rat model of neurogenic myopathy induced by sciatic nerve constriction (SNC).

## Results

### Skeletal muscle proteostasis is impaired during progression of neurogenic myopathy

**Figure 1:**
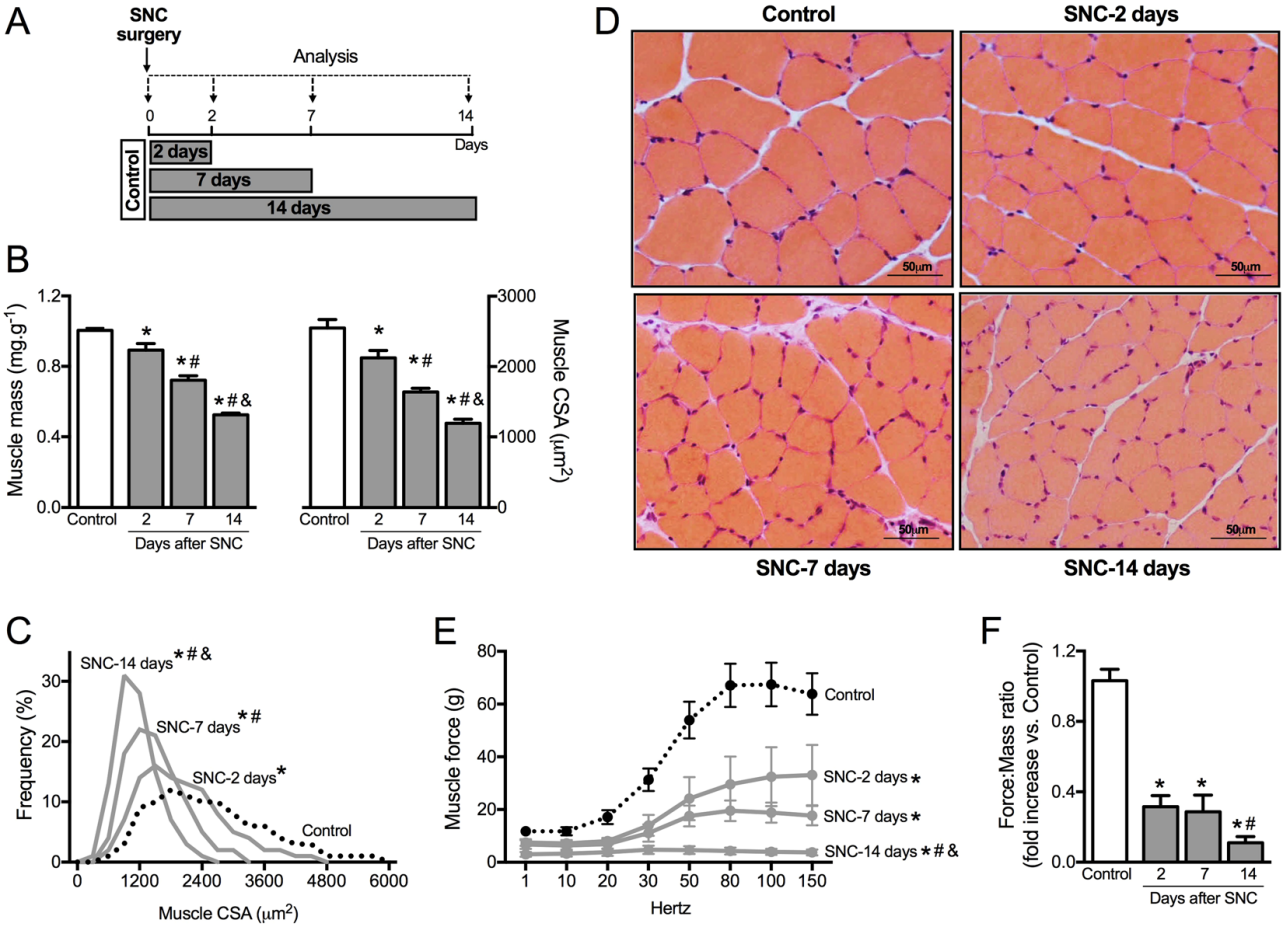
Skeletal muscle morphology and function in SNC-induced neurogenic myopathy. (**A**) Schematic panel of Study Design 1: rats were submitted to SNC or equal procedure without ligation of the sciatic nerve (sham surgery – Control group). In order to follow the disuse-induced skeletal muscle atrophy, morphological and functional parameters were evaluated in skeletal muscle 2, 7 and 14 days after SNC. (**B**) Skeletal muscle mass and myofiber CSA, (**C**) frequency distribution of CSA and (**D**) representative images from plantaris muscle. (**E**) *Ex vivo* skeletal muscle function assessed by development of force in response to stimulus frequencies of 1, 10, 20, 30, 50, 80, 100 and 150 hertz (force-frequency protocol) and (**F**) force: mass ratio in EDL muscle from Control and SNC (2, 7 and 14 days after surgery) rats. Data are presented as mean ± SEM. *p < 0.05 vs. Control; ^#^p < 0.05 vs. SNC-2days; ^&^p < 0.05 vs. SNC-7days; n = 8–12 animals.

Skeletal muscle proteostasis is impaired during progression of neurogenic myopathy. To investigate the skeletal muscle proteostasis profile during progression of neurogenic myopathy, we first measured morphological and functional parameters of skeletal muscle in a rat model of permanent SNC (Fig. 1A). A progressive muscle wasting, depicted by reduced muscle mass, was observed in plantaris muscle following 2, 7 and 14 days of SNC (Fig. 1B). The skeletal muscle atrophy that reached ~55% 14 days after surgery was also apparent at the single fiber level, measured by myofiber cross-sectional area (CSA). Moreover, the distribution of fiber sizes in plantaris muscle showed a leftward shift during disuse due to an increase in the small fibers percentage (Fig. 1B–D). These changes were accompanied by a reduction in extensor digitorum longus muscle (EDL) *ex vivo* contractile properties assessed by maximal tetanic absolute force (Fig. 1E,F) when compared to Control rats.

**Figure 2:**
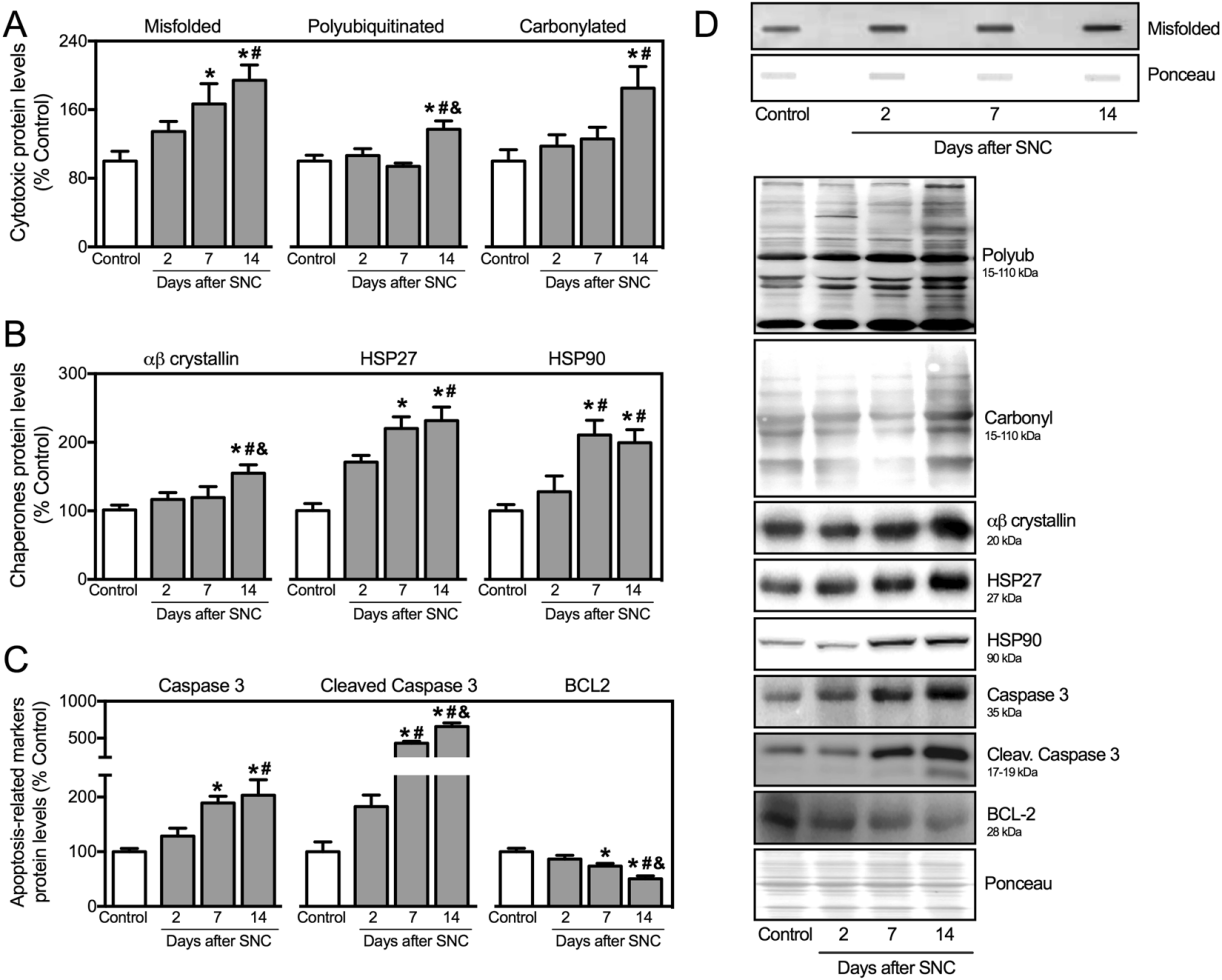
Skeletal muscle proteostasis is impaired during progression of neurogenic myopathy. Protein levels of (**A**) cytotoxic proteins (misfolded, polyubiquitinated and protein carbonyls), (**B**) proteostasis-related markers (αβ-crystallin, HSP27 and HSP90) and (**C**) apoptosis-related markers (caspase 3, cleaved caspase 3 and BCL-2), and (**D**) representative images of plantaris muscle from Control and SNC (2, 7 and 14 days after surgery) rats. The corresponding ponceau stain was used to verify equal loading of proteins and values are expressed as a percentage of the Control group. Misfolded proteins were assessed by slot blot. Data are presented as mean ± SEM. *p < 0.05 vs. Control; ^#^p < 0.05 vs. SNC-2days; ^&^p < 0.05 vs. SNC-7days; n = 8–12 animals.

We next measured the levels of critical markers of skeletal muscle proteostasis in neurogenic myopathy. The progression of muscle atrophy and dysfunction in SNC rats was followed by increased levels of misfolded, polyubiquitinated and carbonylated proteins in skeletal muscle of SNC animals (Fig. 2A,D). Accumulation of damaged proteins was paralleled by elevated levels of chaperones αβ-crystallin, HSP27 and HSP90 in SNC animals over 14 days of neurogenic myopathy (Fig. 2B,D). Finally, the progression of skeletal muscle atrophy and dysfunction was depicted by increased levels of pro-apoptotic markers caspase 3 and cleaved caspase 3 as well as reduced levels of anti-apoptotic protein BCL-2 in skeletal muscle from SNC animals (Fig. 2C,D). Taken together, these findings suggest that proteostasis is impaired during the progression of skeletal muscle dysfunction in a model of neurogenic myopathy in rats.

### Autophagy is compromised in neurogenic myopathy

**Figure 3:**
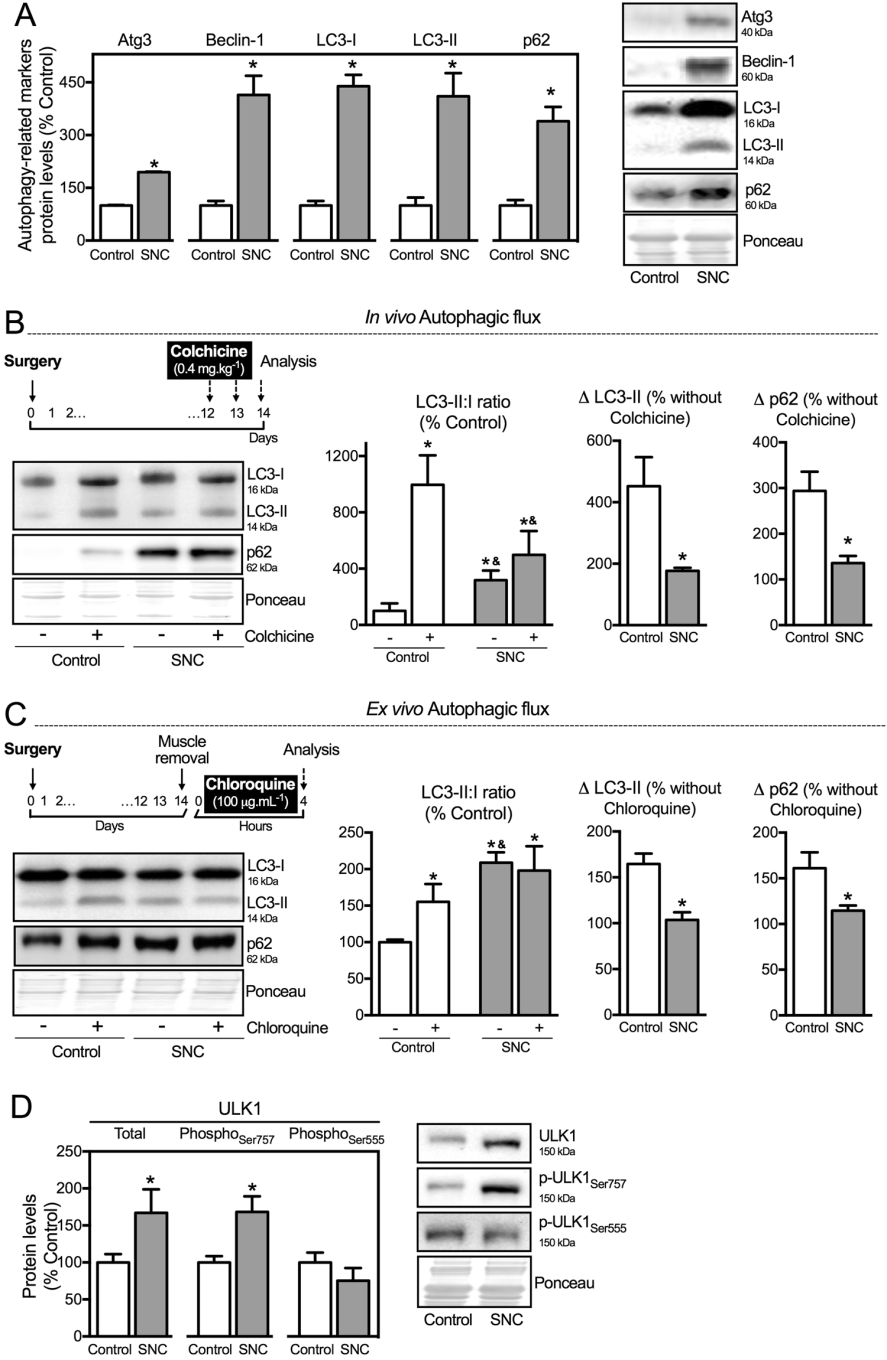
Autophagy is compromised in neurogenic myopathy. (**A**) Protein levels and representative images of autophagy-related markers (Atg3, Beclin – 1, LC3-I, LC3-II and p62). (**B**) Schematic panel of *in vivo* autophagic flux: LC3 and p62 protein levels and representative images of plantaris muscle from rats treated with saline (−) or colchicine (0.4 mg.kg^−1^) (+) 48 and 24 hours before the sacrifice. (**C**) Schematic panel of *ex vivo* autophagic flux: LC3 and p62 protein levels and representative images of plantaris muscle treated *ex vivo* with saline (−) or chloroquine (100 μg.mL^−1^) (+) for 4 hours. (**D**) Protein levels and representative images of ULK1 (Total, phospho-ULK1_Ser757_ and phospho-ULK1_Ser555_) of plantaris muscle from Control and SNC (14 days after surgery) rats. The corresponding ponceau stain was used to verify equal loading of proteins and values are expressed as a percentage of the Control group and as a percentage of the respective group without colchicine or chloroquine (autophagic flux). Data are presented as mean ± SEM. *p < 0.05 vs. Control; n = 8–12 animals.

Considering that autophagy degradation system comprises one of the main effectors in regulating proteostasis^5,24,25^, we set out to determine the skeletal muscle levels of autophagy-related markers as well as the autophagic flux in neurogenic myopathy. At 14 days after SNC there was an increase in autophagy-related markers associated with both elongation and maturation of the autophagosome (Atg3, Beclin-1, LC3 and p62) in atrophic/dysfunctional skeletal muscle compared to Control animals (Fig. 3A). However, due to the dynamism and complexity of autophagy machinery, accumulation of these proteins may reflect either increased or insufficient autophagy process^26^.

To better understand the meaning of increased levels of autophagy-related markers during skeletal muscle atrophy/dysfunction, we carried out two different assays to measure autophagic flux^27,28^. First, we performed an *in vivo* autophagic flux assay by treating the rats 48 hours before the end of the experimental protocol with colchicine (0.4 mg.kg^−1^, a microtubule depolarizing agent that blocks autophagosome maturation to autolysosomes). As expected, colchicine treatment increased skeletal muscle LC3-II:I ratio and LC3-II levels in Control, but not in SNC rats (Fig. 3B). Additionally, we also inhibited autophagic process *ex vivo* by incubating plantaris muscle with chloroquine (100 μg.mL^−1^, 4 h −37 °C, a compound that neutralizes the lysosomal pH), which also resulted in increased LC3-II:I ratio and ~70% accumulation of LC3-II in muscles from Control, but not from SNC animals (Fig. 3C). Both *in vivo* and *ex vivo* autophagic flux assays resulted in accumulation of p62 levels in muscles from Control, but not from SNC rats (Fig. 3B,C). These data suggest that skeletal muscle autophagic flux is compromised/impaired in a model of neurogenic myopathy in rats. Moreover, we evaluated protein levels of ULK1 (Unc-51-like kinases 1) – a protein that governs autophagosome formation^29^. Further supporting autophagy suppression, SNC rats presented increased phosphorylation of ULK1 at Serine 757, a residue targeted by mTORC1 (mechanistic target of rapamycin complex 1), which impairs autophagy^30^. No changes were observed in the phosphorylation of Serine 555, an ULK1 residue that activates autophagy^31^ (Fig. 3D).

SNC rats submitted to chronic inhibition of mTORC1 (I.P. injections of rapamycin 1.5 mg.kg^−1^.day^−1^ for 14 days) displayed decreased levels of phospho-ULK1_Ser757_ along with lower levels of polyubiquitinated proteins and the pro-apoptotic factor CHOP when compared to SNC-saline treated animals (see Supplementary Fig. S1). Decreased levels of phospho-S6_Ser235_ and phospho-4EBP1_Ser65_, associated with less incorporation of puromycin, confirmed the effectiveness of the *in vivo* rapamycin treatment-induced mTORC1 inhibition in SNC rats (see Supplementary Fig. S1). These findings reinforce the hypothesis that constitutive mTORC1 activation contributes to autophagy suppression in skeletal muscle weakness/wasting. However, we cannot exclude the possible role of mTORC1 in other downstream effectors^32^ that regulate proteostasis and skeletal myopathy.

**Figure 4:**
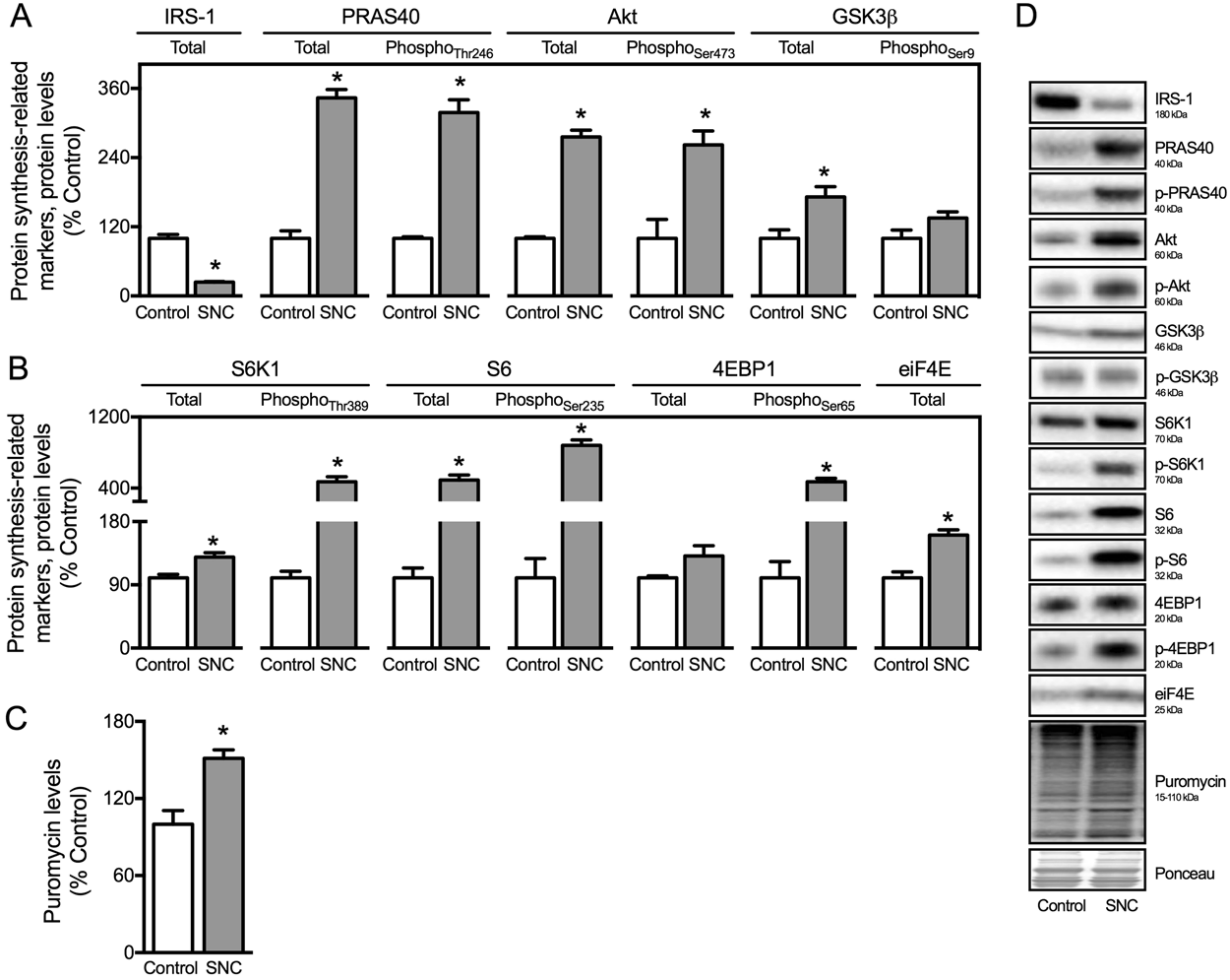
Skeletal muscle protein synthesis is increased in SNC-induced neurogenic myopathy. (**A**,**B**) Protein levels of protein synthesis-related markers (IRS-1, PRAS40, phospho-PRAS40_Thr246_, Akt, phospho-Akt_Ser473_, GSK3β, phospho-GSK3β_Ser9_, S6K1, phospho-S6K1_Thr389_, S6, phospho-S6_Ser235_, 4EBP1, phospho-4EBP1_Ser65_ and eiF4E), (**C**) protein synthesis measured by the SUnSET method (puromycin levels) and (**D**) representative images of plantaris muscle from Control and SNC (14 days after surgery) rats. The corresponding ponceau stain was used to verify equal loading of proteins and values are expressed as a percentage of the Control group. Data are presented as mean ± SEM. *p < 0.05 vs. Control; n = 8–12 animals.

Considering that proteostasis is regulated by both protein synthesis and degradation, we next decided to investigate the protein synthesis profile in skeletal muscle from SNC and Control rats. Skeletal muscle atrophy and dysfunction in SNC rats were followed by increased total and phosphorylated levels of protein synthesis-related markers PRAS40, phospho-PRAS40_Thr246_, Akt and phospho-Akt_Ser473_ (Fig. 4A,D). Moreover, we detected increased levels of protein synthesis downstream effectors S6, phospho-S6_Ser235_, phospho-4EBP1_ser65_ and eiF4E in SNC rats compared to Controls (Fig. 4B,D). Next, to understand whether elevated levels of protein synthesis-related markers reflected into newly synthesized proteins in skeletal muscle from SNC rats, we measured protein synthesis using the *in vivo* incorporation of puromycin – SUnSET method. In fact, puromycin incorporation into skeletal muscle was elevated by about 50% in SNC rats compared with Controls (Fig. 4C,D). Overall, these findings demonstrate that proteostasis imbalance is characterized by increased protein synthesis and reduced autophagy-mediated protein degradation in neurogenic myopathy.

### Chronic autophagy disruption reduces skeletal muscle strength in sham animals

**Figure 5:**
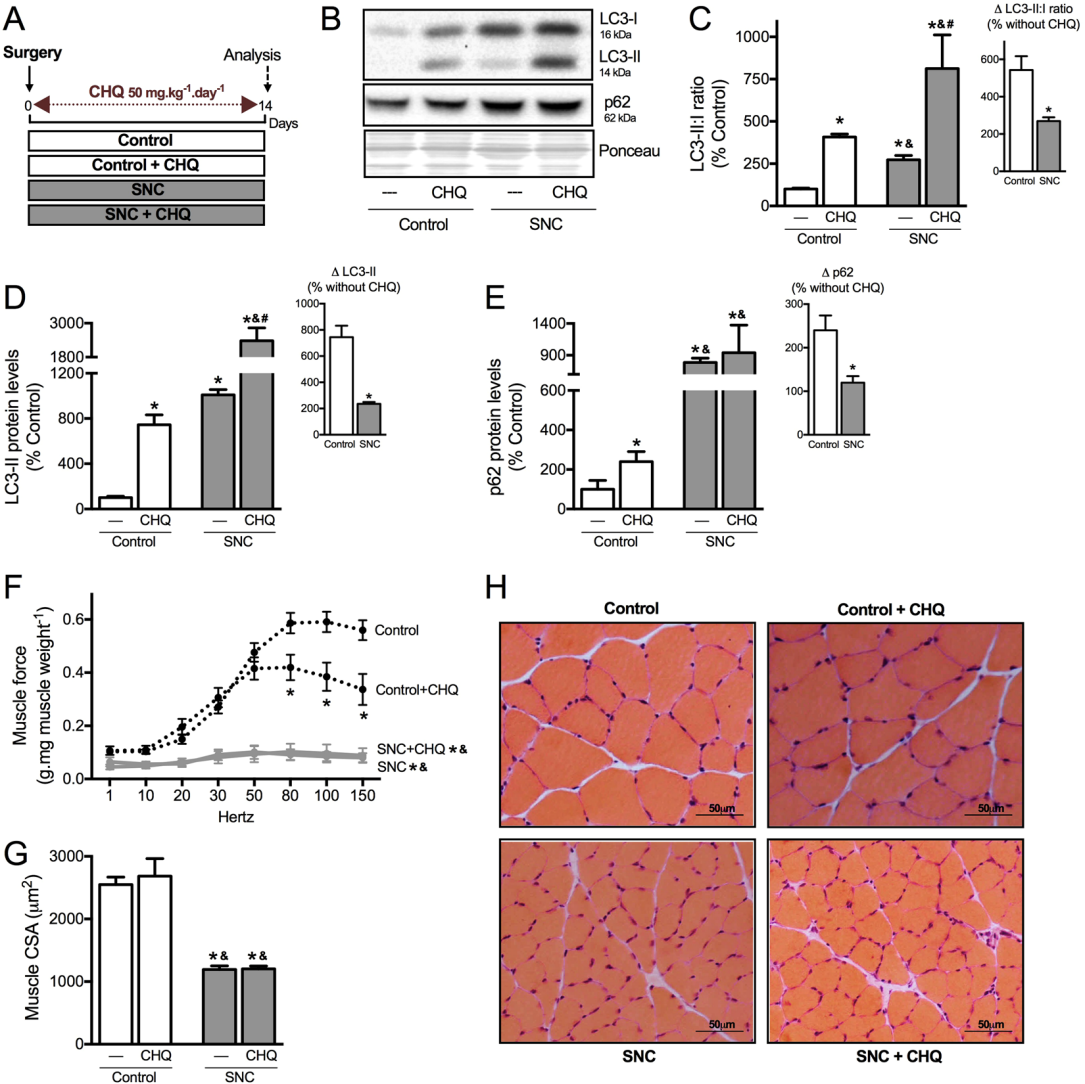
Chronic autophagy disruption *in vivo* reduces skeletal muscle strength in healthy animals, but not in neurogenic myopathy. (**A**) Schematic panel of Study Design 2: rats were submitted to sham or SNC and randomly assigned into saline or chloroquine (CHQ – 50 mg.kg^−1^.day^−1^) treatment groups. At the end of experimental protocol, skeletal muscle morphological, functional and biochemical analyses were performed in Control, Control + CHQ, SNC and SNC + CHQ rats. (**B**) Representative images of LC3 and p62 in plantaris muscle. (**C**) LC3-II:I ratio (input, ∆LC3-II:I ratio after CHQ), (**D**) LC3-II (input, ∆LC3-II after CHQ) and (**E**) p62 (input, ∆p62 after CHQ) protein levels in plantaris muscle. The corresponding ponceau stain was used to verify equal loading of proteins and values are expressed as a percentage of the Control group. (**F**) *Ex vivo* skeletal muscle function assessed by development of force in response to stimulus frequencies of 1, 10, 20, 30, 50, 80, 100 and 150 hertz (force-frequency protocol) in EDL muscle. Forces are expressed in grams and normalized by the EDL muscle wet weight. (**G**) Myofiber CSA and (**H**) representative images of plantaris muscle from Control, Control + CHQ, SNC and SNC + CHQ rats. Data are presented as mean ± SEM. *p < 0.05 vs. Control; ^&^p < 0.05 vs. Control + CHQ; ^#^p < 0.05 vs. SNC; n = 8–12 animals.

Next, we decided to evaluate whether chronic inhibition of autophagy is sufficient to impair skeletal muscle contractile properties and/or trophism. Autophagy was chronically inhibited by treating Control (sham) and SNC rats daily with chloroquine (50 mg.kg^−1^.day^−1^) for 14 days (Fig. 5A). As expected, sustained chloroquine inhibition resulted in a 5- and 7-fold increase in LC3-II:I ratio and LC3-II levels, respectively, in skeletal muscle from control animals; therefore confirming the effectiveness of our treatment (Fig. 5B–D). Treatment of SNC animals with chloroquine resulted in a mild increase of LC3-II:I ratio and LC3-II levels in skeletal muscle (~2-fold, Fig. 5B–D). According to the *Guidelines for the use and interpretation of assays monitoring autophagy*^26^, this increase in LC3-II levels after chronic treatment with chloroquine does not reflect the autophagic flux. Additionally, chronic chloroquine treatment induced an accumulation of p62 in Control group, but not in SNC (Fig. 5B,E).

Chronic pharmacological inhibition of autophagy was able to reduce *ex vivo* skeletal muscle contractility properties (Fig. 5F) without affecting trophism (Fig. 5G,H) in Control rats. Chloroquine treatment did not aggravate skeletal muscle wasting or dysfunctional contractile properties in SNC rats when compared to non-treated SNC group (Fig. 5F–H). Considering that chloroquine blocks not only autophagy, but the whole lysosomal degradation pathway, we decided to validate these findings using Atg7 KO mice. Muscle specific deletion of Atg7 was sufficient to reduce muscle contractility properties in Control but not in SNC mice, without affecting trophism (Supplementary Fig. S2). These findings using pharmacological inhibition (chloroquine) and genetic disruption (Atg7 KO mice) of autophagy reinforce the critical role of autophagy in regulating skeletal muscle contractility properties and suggest that autophagy is already compromised in a model of neurogenic myopathy in rats.

### Exercise training activates autophagy and improves proteostasis in neurogenic myopathy

**Figure 6:**
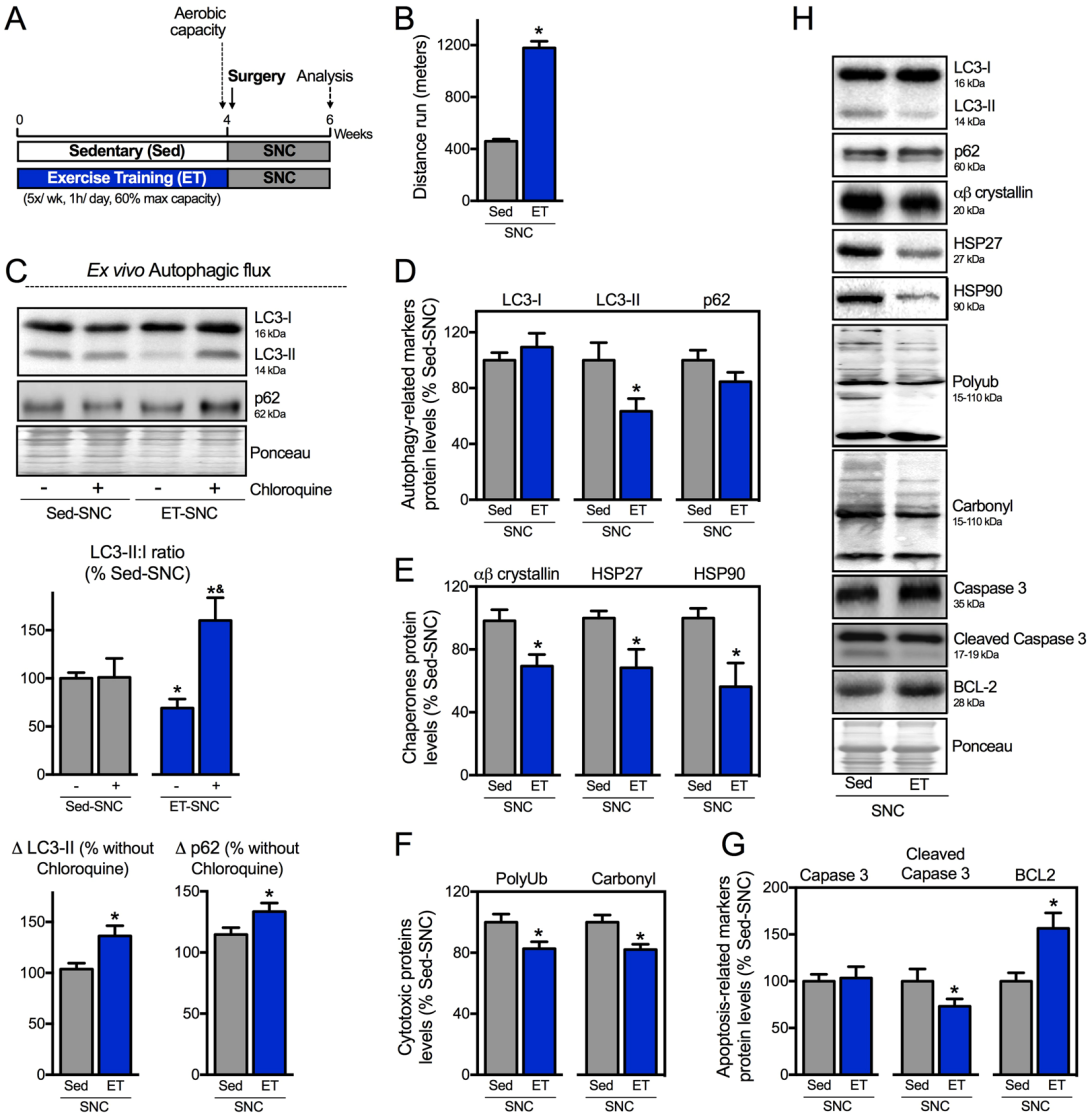
Exercise training activates autophagy and improves proteostasis in neurogenic myopathy. (**A**) Schematic panel of Study Design 3: rats were randomly assigned into sedentary (Sed) and exercise training (ET) groups. ET rats were submitted to running on a treadmill over 4 weeks, 5 days/week, 60 minutes per day at 60% of maximal aerobic capacity. After this period rats were submitted to SNC, and 14 days after surgery skeletal muscle morphological, functional and biochemical parameters were measured in Sed-SNC and ET-SNC rats. (**B**) Aerobic capacity evaluated by total distance run after protocol (week 4). (**C**) *Ex vivo* autophagic flux: LC3 and p62 protein levels and representative images of plantaris muscle from Sed-SNC and ET-SNC rats treated *ex vivo* with saline (−) or chloroquine (100 μg.mL^−1^) (+) for 4 hours. Protein levels of (**D**) autophagy-related markers (LC3-I, LC3-II and p62), (**E**) chaperones (αβ-crystallin, HSP27 and HSP90), (**F**) cytotoxic proteins (polyubiquitinated and protein carbonyls) and (**G**) apoptosis-related markers (caspase 3, cleaved caspase 3 and BCL-2), and (**H**) representative images of plantaris muscle from Sed-SNC and ET-SNC rats. The corresponding ponceau stain was used to verify equal loading of proteins and values are expressed as a percentage of the Sed-SNC group and as a percentage of the respective group without chloroquine (autophagic flux). Data are presented as mean ± SEM. *p < 0.05 vs. Sed-SNC; n = 8–12 animals.

Considering that autophagy is impaired in neurogenic myopathy, we next set out to determine whether the re-establishment of autophagic flux is sufficient to improve skeletal muscle proteostasis and counteract SNC-induced skeletal muscle damage. We have recently demonstrated that exercise training is able to restore the autophagic flux in failing hearts contributing to better disease prognosis^21^. To evaluate the effects of exercise training in autophagy in a model of neurogenic myopathy, rats were submitted to 4 weeks of moderate running training on a treadmill prior to SNC surgery. Skeletal muscle trophism, function, autophagy and proteostasis were evaluated 14 days after surgery in sedentary (Sed-SNC) and trained SNC (ET-SNC) rats (Fig. 6A).

We demonstrated that 4 weeks of exercise training was sufficient to improve aerobic capacity (Fig. 6B). To test the effectiveness of exercise training in activating skeletal muscle autophagy we performed an *ex vivo* autophagic flux assay. Exercise increased skeletal muscle responsiveness to acute chloroquine treatment in SNC rats, depicted here by a chloroquine-induced increase in LC3-II:I ratio and an accumulation of both LC3-II and p62 when compared to non-exercised SNC group (Fig. 6C). These findings provide evidence that 4 weeks of exercise prior to SNC is sufficient to increase autophagic flux in a rat model of neurogenic myopathy.

**Figure 7:**
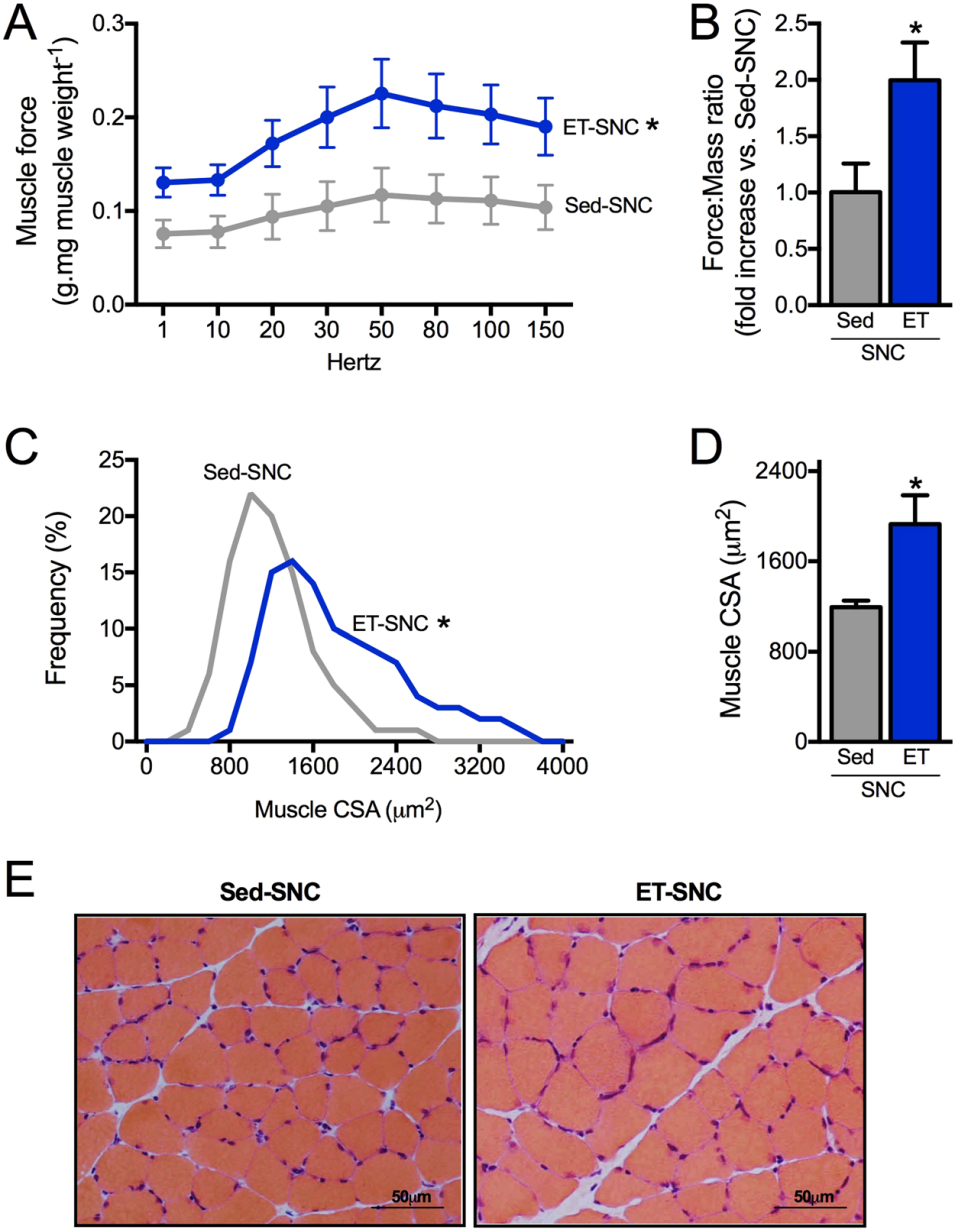
Positive effect of exercise training on skeletal muscle mass and function in neurogenic myopathy. (**A**) *Ex vivo* skeletal muscle function assessed by development of force in response to stimulus frequencies of 1, 10, 20, 30, 50, 80, 100 and 150 hertz (force-frequency protocol) and (**B**) force: mass ratio in EDL muscle. Forces are expressed in grams and normalized by the EDL muscle wet weight. (**C**) Frequency distribution of CSA, (**D**) myofiber CSA and (**E**) representative images of plantaris muscle from Sed-SNC and ET-SNC rats. Data are presented as mean ± SEM. *p < 0.05 vs. Sed-SNC; n = 8–12 animals.

Activation of autophagy was associated with reduced levels of skeletal muscle LC3-II (Fig. 6D,H), polyubiquitinated and carbonylated proteins in exercised SNC animals compared to sedentary group (Fig. 6F,H). These changes in proteostasis were followed by reduced cellular stress in skeletal muscle, depicted here by decreased levels of chaperones αβ-crystallin, HSP27 and HSP90 (Fig. 6E,H) as well as decreased levels of cleaved caspase 3 (a pro-apoptotic related marker), and increased levels of BCL2 (an anti-apoptotic related marker) (Fig. 6G,H), in exercised animals compared with sedentary group 14 days after SNC. Finally, we provided evidence that increased skeletal muscle autophagic flux and proteostasis induced by exercise training was accompanied by better *ex vivo* skeletal muscle contractile properties (Fig. 7A,B) as well as increased skeletal muscle fiber CSA compared to sedentary SNC animals (Fig. 7C–E).

## Discussion

Maintenance of skeletal muscle functionality is critical for healthy ageing and has a positive impact on quality of life and survival in many degenerative diseases^18^. However, the spectrum of pharmacological and non-pharmacological interventions capable of protecting skeletal muscle is still limited. Therefore, identifying cellular and molecular mechanisms that counteract skeletal myopathy/dysfunction must be extensively studied.

Here, using an *in vivo* model of neurogenic myopathy, we provided evidence that dysfunctional/atrophic muscles exhibit impaired proteostasis, characterized by accumulation of damaged and stress-related proteins. Theses changes were associated with an imbalance between protein synthesis and degradation, depicted by increased protein synthesis and reduced autophagic flux. Further supporting an autophagic mechanism for impaired skeletal muscle functionality, pharmacological inhibition of autophagy was sufficient to reduced skeletal muscle contractility properties in Control animals. Finally, we demonstrated that exercise training improved skeletal muscle autophagic flux and proteostasis in neurogenic myopathy, which was associated with spared muscle mass and better contractility properties.

Skeletal muscle degeneration is commonly associated with impaired protein synthesis and excessive protein degradation; therefore resulting in muscle atrophy with impact on contractile properties^33^. Here, we observed that chronic neurogenic myopathy is paradoxically characterized by increased protein synthesis 14 days after muscle denervation. Elevated protein synthesis has been previously reported in an acute model of skeletal muscle denervation, which was associated with the maintenance of skeletal muscle mass^34^. The elevation in protein synthesis seems to be a compensatory mechanism to counteract the excessive muscle loss in neurogenic myopathy. However, the mechanisms related to this process as well as its contribution in maintaining skeletal muscle mass and functionality in chronic degenerative diseases are still elusive.

Excessive protein degradation is a hallmark of skeletal muscle atrophy^3,35–40^. Different genetic and pharmacological interventions that counteract exacerbated proteolysis seem to be sufficient to protect against muscle atrophy in preclinical studies^37,41–43^. However, it is expected that uncontrolled inhibition of proteolysis causes proteotoxicity, an adverse effect that can impair muscle contractility properties in the mid-to-long term^44^. In fact, we and others have demonstrated that inhibition of proteolysis results in increased cardiotoxicity in both rodents^7^ and humans^45^. Moreover, genetic inclusion body myopathy, which is a severe proteinopathy, causes a progressive skeletal muscle weakness and atrophy in both rodents and humans^17^. Therefore, the maintenance of proteostasis through different interdependent systems ultimately dictates cell and tissue viability. A sustained disruption in protein homeostasis caused by impaired protein degradation and consequent accumulation of dysfunctional proteins is sufficient to trigger cell death^6,7^. We recently reported that improving the turnover of toxic misfolded proteins^7,20,46–48^, as well as mal-functioning organelles^21^ results in better cardiac outcome during degenerative processes.

It is well known that autophagy plays a critical role in the clearance of damaged proteins, therefore affecting skeletal muscle physiology^49^. Evidence supporting the detrimental role of dysfunctional autophagy in skeletal muscle pathophysiology emerged from biochemical, functional and ultrastructural analysis in muscle-specific autophagy-deficient mice. Two different groups independently demonstrated that genetic disruption of autophagic flux contributes to myofiber degeneration and skeletal muscle weakness in rodents^15,50^. Moreover, skeletal muscle autophagy is suppressed in acute denervation^34^. Here, we provide evidence that skeletal muscle accumulation of dysfunctional/cytotoxic proteins occurs in parallel to impaired autophagy in chronic neurogenic myopathy. Indeed, sustained inhibition of autophagy using chloroquine, a chemical compound that neutralizes lysosomal pH, is sufficient to impair skeletal muscle contractility properties in control but not in neurogenic myopathy animals. Together, these data suggest that loss of autophagic flux plays a detrimental role in the maintenance of skeletal muscle protein homeostasis, which contributes to the pathophysiology of chronic neurogenic myopathy. In fact, genetic diseases characterized by impaired autophagy and proteinopathy commonly causes severe skeletal myopathy^5,17^.

Restoring skeletal muscle autophagy ameliorates the dystrophic phenotype in a variety of conditions^14,49,51–54^. Exercise has been used as a non-pharmacological intervention capable of activating autophagy (acutely and chronically) in different tissues^21,55,56^. Although the contribution of skeletal muscle autophagy to exercise performance is still controversial^23,57,58^, exercise-induced autophagy in degenerative diseases plays a critical role in facilitating the turnover of damaged proteins and dysfunctional organelles (i.e. mitochondria)^21,59^. Here, we found that 4 weeks of exercise training, starting prior to SNC surgery as a preventive intervention, was sufficient to improve skeletal muscle autophagic flux, which was associated with reduced levels of dysfunctional/cytotoxic protein and markers of apoptosis in chronic neurogenic myopathy. Overall, these changes contributed to the maintenance of skeletal muscle protein homeostasis, which partially affected muscle trophism and contractility. These data indicate that autophagic flux is unlikely the only factor that affects neurogenic myopathy. In fact, our data using chronic chloroquine treatment provide evidence that autophagy reduced muscle force in a lower magnitude in control animals comparing with untreated SNC rats. Despite of autophagy contribution to skeletal myopathy, its degenerative process is associated with other intracellular events such as impaired calcium handling, redox status and mitochondrial metabolism, which are positively affected by exercise^60–64^. In conclusion, our findings suggest that skeletal muscle protein homeostasis is impaired in an animal model of chronic neurogenic myopathy, most likely resulting from defective autophagy. Our data also suggest that exercise training may become a useful strategy to prevent disruption of skeletal muscle proteostasis in chronic neurogenic myopathy.

## Materials and Methods

### Study Design

1. A cohort of male Sprague-Dawley rats was submitted to SNC or equal procedure without ligation of the sciatic nerve (sham surgery – Control group). In order to follow the SNC-induced skeletal muscle atrophy, morphological, functional and biochemical parameters were evaluated in skeletal muscle 2, 7 and 14 days after surgery (Figs 1 and 2). At the end of the experimental protocol, protein degradation and protein synthesis were assessed in Control and SNC rats (Figs 3 and 4).
2. Rats were submitted to sham or SNC surgery and randomly assigned into saline or chloroquine (CHQ) treatment groups. To inhibit autophagy *in vivo*, rats were treated daily for 14 days with I.P. injections of CHQ (50 mg.kg^−1^.day^−1^). At the end of experimental protocol, skeletal muscle morphological, functional and biochemical analyses were performed in Control, Control + CHQ, SNC and SNC + CHQ rats (Fig. 5).
3. Rats were randomly assigned into sedentary (Sed) and exercise training (ET) groups. ET rats were submitted to running on a treadmill over 4 weeks, 5 days per week, 60 minutes per day at 60% of maximal aerobic capacity. After this period, animals were submitted to SNC, and 14 days after surgery skeletal muscle morphological, functional and biochemical parameters were measured in Sed-SNC and ET-SNC rats (Figs 6 and 7).

### Animal care and use

A cohort of male Sprague-Dawley rats (250–300 g) was selected for the study. Rats were maintained in a 12:12 h light-dark cycle and temperature-controlled environment (22 °C) with free access to standard laboratory chow and tap water. At the end of protocol, 14 days after sham or SNC surgery rats were anaesthetized using isoflurane [4% (v/v) induction, 3% (v/v) maintenance] and killed by decapitation. The animal care and protocols in this study were reviewed and approved by the Ethical Committee of Institute of Biomedical Sciences of University of Sao Paulo (2012/142/135/02), and were performed in compliance with the National Institutes of Health *Guidelines for the Care and Use of Laboratory Animals*.

### Sciatic nerve constriction (SNC) surgery

For induction of skeletal muscle atrophy, SNC was performed as previously described^65^. Rats were anesthetized with a cocktail of ketamine (50 mg.kg^−1^) and xylazine (10 mg.kg^−1^) and both corneal and toepinch reflex were tested to ensure the adequate depth of anesthesia has been attained. A 0.5-cm incision at middle thigh level on the lateral side of the right hindlimb was made and after blunt dissection through the biceps femoris, the sciatic nerve was lifted out with surgical forceps and four ligatures (4.0 chromic gut) were tied loosely around it. The suture thread causes nerve compression and triggers an immune response causing intraneural edema, effectively axotomizing many but not all of the nerve axons (Wallerian degeneration). This impaired neural input severely compromises skeletal muscle mass and function. Surgery with equal procedure duration to that of SNC group, but without sciatic nerve ligation, was undertaken in the Control group (sham-operated).

### Chloroquine treatment

In order to inhibit the autophagy machinery, rats were treated during 14 days with daily I.P. injections containing chloroquine (50 mg.kg^−1^). A similar volume of saline was injected as control.

### Graded treadmill exercise test

Animals from Study 3 were submitted to graded exercise testing on a motor treadmill adapted to experimental models before and after the experimental period. After being adapted to treadmill exercises and the test environment for over one week (10 minutes each session), rats were placed in the treadmill streak and allowed to acclimatize for at least 30 minutes. Treadmill speed started at 6 m.min^−1^ and was increased by 3 m.min^−1^ every 3 minutes at 0% grade until exhaustion, where rat could no longer maintain running speed over 3 minutes. This test provided the total distance run and peak workload was measured at the termination of the test^66^.

### Exercise training protocol

ET + SNC rats performed moderated-intensity running training on a motor treadmill over 4 weeks, 5 days per week, 60 minutes per day prior to SNC. Running speed and duration of exercise were progressively increased to elicit 60% of maximal speed (corresponding to the maximal lactate steady state workload) at the second week of training^66^.

### Measurement of autophagic flux

*In Vivo*. Autophagic flux was measured *in vivo* as described elsewhere^27^. Control and SNC rats were treated 48 hours before the sacrifice with I.P. injections containing colchicine (0.4 mg kg^−1^). The differences in LC3-II:I ratio, LC3-II and p62 protein levels with and without colchicine – assessed in plantaris muscle by Western Blotting, were used to calculate the autophagic flux. LC3-II:I ratio was expressed as a percentage of Control group and LC3-II and p62 levels were expressed as a percentage of each group without colchicine (arbitrarily set as 100%).

*Ex Vivo*. Autophagic flux was measured *ex vivo* using a modified protocol^67,68^. At the end of the protocol, rats were sacrificed and plantaris muscle was dissected in small chunks. The small chunks were minced finely and then divided between two wells (with or without chloroquine – 100 μg.mL^−1^) and then incubated at 37 °C for 4 h. The differences in LC3-II:I ratio, LC3-II and p62 protein levels with and without chloroquine – assessed in small plantaris chunks by Western Blotting, were used to calculate the autophagic flux. LC3-II:I ratio was expressed as a percentage of Control group and LC3-II and p62 levels were expressed as percentage of each group without chloroquine (arbitrarily set as 100%).

### *Ex vivo* skeletal muscle function

Contractile properties of the fast-twitch EDL muscles were evaluated *ex vivo* as previously described^69^. Briefly, the entire operated hindlimb was removed, the EDL muscle was then carefully isolated and transferred to a temperature-controlled organ bath containing 20 mL of Tyrode solution (137 mM NaCl; 24 mM NaHCO_3_; 5 mM KCl, 2 mM CaCl_2_, 1 mM KH_2_PO_4_, 1 mM MgSO_4_, 11 mM glucose, pH 7.4) at 25 °C bubbled continuously with carbogen (95% O_2_ + 5% CO_2_). The distal tendon was tied to a holder fixed in the organ bath and the proximal tendon was attached to a force transducer (Grass Instruments model FT03, USA). After being mounted, muscle optimal length (L0) was determined from micromanipulations of muscle length and a series of isometric twitch contractions. The muscles were stimulated to contract isometrically using electrical field stimulation (Grass Instruments S-88 Grass-stimulator) delivered via two platinum wire electrodes. The output of the force transducer was recorded and analyzed using a PowerLab system (AD Instruments, USA). To define force-frequency characteristics, we measured force in response to stimulus frequencies of 1, 10, 20, 30, 50, 80, 100 and 150 hertz every 3 minutes. Forces are expressed in grams and normalized by the EDL weight.

### Skeletal Muscle Morphology (CSA)

To evaluate muscle atrophy, fast-twitch plantaris muscle was excised, snap frozen in melting isopentane and stored in liquid nitrogen. Muscles were vertically mounted at L0 length in fixed bases and serially sectioned in cryostat (10 µm sections). Sections were submitted to Hematoxylin-eosin staining for examination by light microscopy. To determine myofiber CSA (µm^2^), images were evaluated at 200 magnification and analyzed by a digitalizing unit connected to a computer (Image Pro-Plus, NHI, USA). A total of approximately 400 myofibers per muscle was measured. A single observer, who was blinded to animal identity, conducted all analyses.

### Protein synthesis measurement

Protein synthesis was measured *in vivo* in rats using the SUnSET method as previously described^70^. Exactly 30 minutes before the plantaris muscles were excised, mice were given an I.P. injection of puromycin (0.04 μmol.g^−1^) dissolved in 100 μl of phosphate buffered saline. Skeletal muscle puromycin levels were detected by Western Blotting and expressed as percentage of Control group (arbitrarily set as 100%).

### Skeletal muscle protein levels (western blotting)

Protein levels were measured by western blotting in total lysate from plantaris muscle. Briefly, samples were subjected to SDS-PAGE in polyacrylamide gels (6–15%) depending upon protein molecular weight. After electrophoresis, proteins were electrotransferred to PVDF membranes. Equal gel loading and transfer efficiency were monitored using 0.5% Ponceau S staining of blot membrane. Blotted membrane was then blocked in 5% nonfat dry milk T-TBS (10 mM Tris-HCl (pH = 7.6), 150 mM NaCl, and 0.1% Tween 20) for 2 hours at room temperature and then incubated overnight at 4 °C with specific antibodies against polyubiquitinated conjugates, αβ-crystallin, HSP27, HSP90, BCL-2, caspase 3, cleaved caspase 3, Atg3, Beclin-1, LC3, p62/SQSTM1, ULK1, phospho-ULK1_Ser757_, phospho-ULK1_Ser555_, PRAS40, phospho-PRAS-40_Thr246_, Akt, phospho-Akt_Ser473_, GSK3β, phospho-GSK3β_Ser9_, S6K1, phospho-S6K1_Thr389_, 4EBP1, phospho-4EBP1_Ser65_, eIF4E and IRS-1. Binding of the primary antibody was detected with the use of peroxidase-conjugated secondary antibodies (rabbit, mouse or goat, depending on the protein, for 2 hours at room temperature) and developed using enhanced chemiluminescence detected by autoradiography. Quantification analysis of blots was performed with the use of Scion Image software (Scion based on NIH image). Results were corrected to Ponceau red staining (0.5%, w:v) of the membrane and expressed as percentage of Control or Sed-SNC groups (arbitrarily set as 100%).

### Misfolded protein levels

Upon conformational changes, misfolded proteins acquire a motif known as the beta-sheet and often expose beta-strand oligomers. This new structure confers ability to self-assemble with other misfolded proteins (protein aggregation)^71,72^. Beta-strand (cellular soluble) oligomers can be recognized by an antibody in a manner that is independent of amino acid sequence^72^. Briefly, to assess the levels of misfolded proteins, 25 μg of plantaris muscle protein samples were slot-blotted onto PVDF membrane using a Minifold II slot blot apparatus. The membranes were washed 3X with T-TBS and then blocked for 1 hour in 5% nonfat dry milk T-TBS. After blocking, blots were incubated overnight at 4 °C with anti-soluble oligomer. Blots were then incubated with a secondary anti-IgG rabbit antibody linked to horseradish peroxidase for 2 hours. Protein slots were visualized and quantified using the Scion Image software. Results were corrected to Ponceau red staining (0.5%, w:v) of the membrane and expressed as percentage of Control group (arbitrarily set as 100%).

### Carbonylated protein levels

Protein carbonylation was assessed by measuring the levels of carbonyl groups using the OxyBlot Protein Detection Kit from Millipore, as previously described^73^. Briefly, 20 μg of soluble proteins were denatured by 6% SDS (w:v) and the carbonyl groups in the protein side chains were derivatized to 2,4-dinitrophenylhydrazone (DNP) by reaction with 2,4-dinitrophenylhydrazine (DNPH). The reaction was stopped and the skeletal muscle protein carbonylation was detected by Western Blotting, quantified in the broadest molecular weight range as possible and expressed as percentage of Control or Sed-SNC groups (arbitrarily set as 100%).

### Statistical analysis

Data are presented as means ± standard error of the mean (SEM). Data normality was assessed through Shapiro-Wilk’s test. One-way analysis of variance (ANOVA) was used to analyze data from Figs 1, 2, 6 and 7. Two-way ANOVA was used to analyze data from Fig. 5. Whenever significant F-values were obtained, Duncan’s adjustment was used for multiple comparison purposes. Student t test was used to analyze data from Figs 3 and 4. Statistical significance was considered achieved when the value of P was < 0.05. Kolmogorov–Smirnov test was applied in order to test differences between groups in Figs 1C and 7C.

### Data availability

The datasets generated and/or analyzed during the current study are available from the corresponding author.

## Electronic supplementary material

Supplementary information
