# Supplementary material for: Exercise prevents impaired autophagy and proteostasis in a model of neurogenic myopathy
Source: Sci Rep. 2018 Aug 7;8:11818. doi: 10.1038/s41598-018-30365-1 (PMC6081439; doi:10.1038/s41598-018-30365-1)
Supplement: Supplementary file 1 — Supplementary information [file 41598_2018_30365_MOESM1_ESM.doc]

**Supplementary Information**:

**Exercise prevents impaired autophagy and proteostasis in a model of neurogenic myopathy**

**Juliane C. Campos^1^, Leslie M. Baehr^2^, Kátia M.S. Gomes^1^, Luiz R.G. Bechara^1^, Vanessa A. Voltarelli^3^, Luiz H.M. Bozi^1^, Márcio A.C. Ribeiro^1^, Nikolas D. Ferreira^1^, José B.N. Moreira^3,4^, Patricia C. Brum^3^, Sue C. Bodine^2^, and Julio C.B. Ferreira^1,*^**

^1^Institute of Biomedical Sciences, University of Sao Paulo, Sao Paulo, 05508-000, Brazil

^2^Department of Internal Medicine, Endocrinology and Metabolism Division, University of Iowa, Iowa, 52242, USA

^3^School of Physical Education and Sport, University of Sao Paulo, Sao Paulo, 05508-030, Brazil

^4^Cardiac Exercise Research Group, Faculty of Medicine and Health Sciences, Norwegian University of Science and Technology, Trondheim, 7006, Norway

**^*^**jcesarbf@usp.br


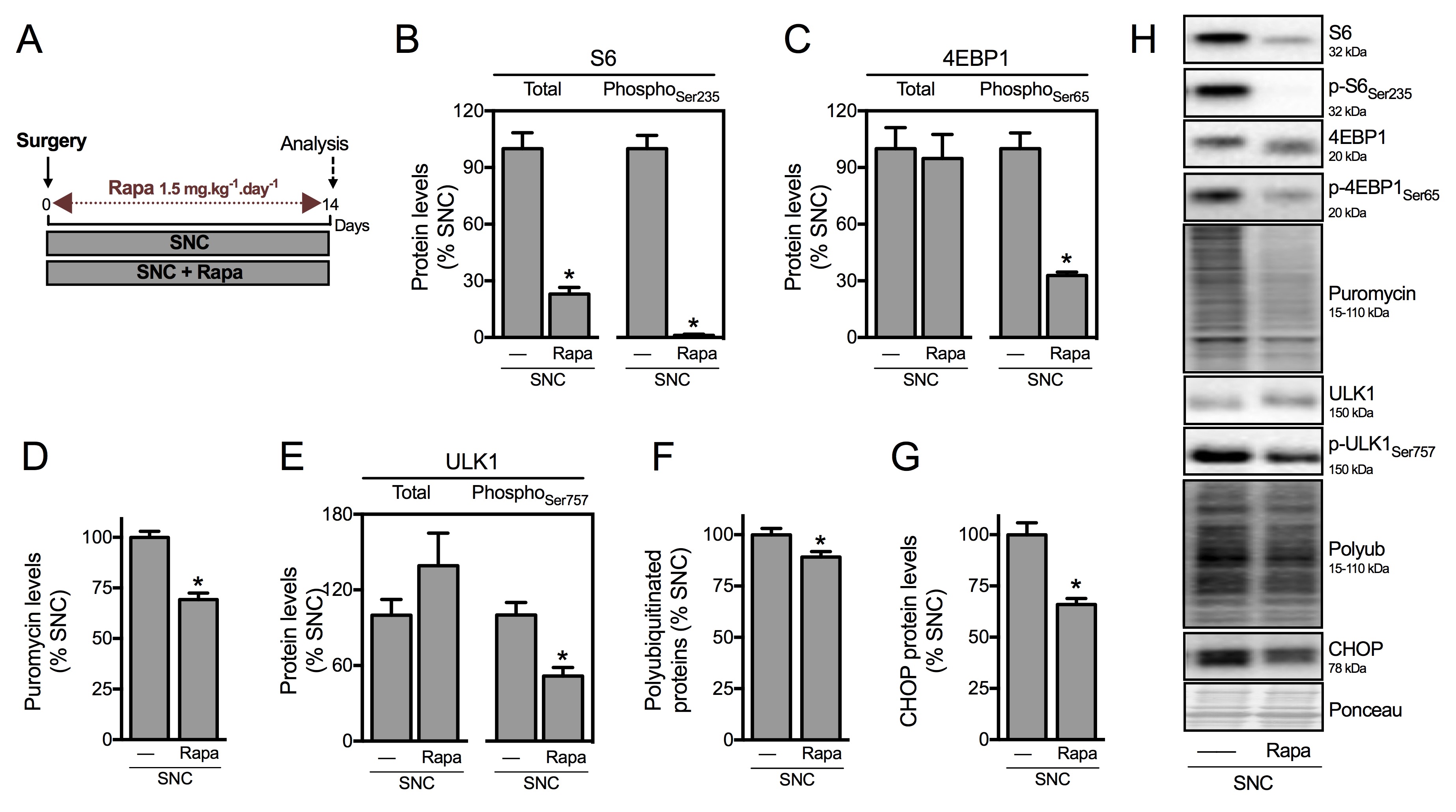


**Supplementary Figure S1.** *In vivo* mTORC1 inhibition-induced autophagy improves proteostasis in neurogenic myopathy. (**A**) Schematic panel: rats were submitted to SNC and randomly assigned into saline or rapamycin treatment groups. In order to inhibit mTORC1, rats were treated during 14 days with daily I.P. injections containing rapamycin (Rapa – 1.5 mg.kg^-1^.day^-1^) starting the day of the surgery. A similar volume of saline was injected as control. At the end of experimental protocol, skeletal muscle biochemical analyses were performed in SNC and SNC+Rapa rats. (**B** and **C**) Protein levels of mTORC1 substrates S6, phospho-S6_Ser235_, 4EBP1 and phospho-4EBP1_Ser65_, (**D**) protein synthesis measured by the SUnSET method (puromycin levels), (**E**) protein levels of ULK1 and phospho-ULK1_Ser757_, (**F**) polyubiquitinated proteins, (**G**) protein levels of CHOP and (**H**) representative images of plantaris muscle from SNC and SNC+Rapa rats. The corresponding ponceau stain was used to verify equal loading of proteins and values are expressed as a percentage of the SNC group. Data are presented as mean ± SEM. Data normality was assessed through Shapiro-Wilk’s test. Student t test was applied and statistical significance was considered achieved when the value of P was < 0.05. *, p<0.05 vs. SNC; n=5-6 animals.


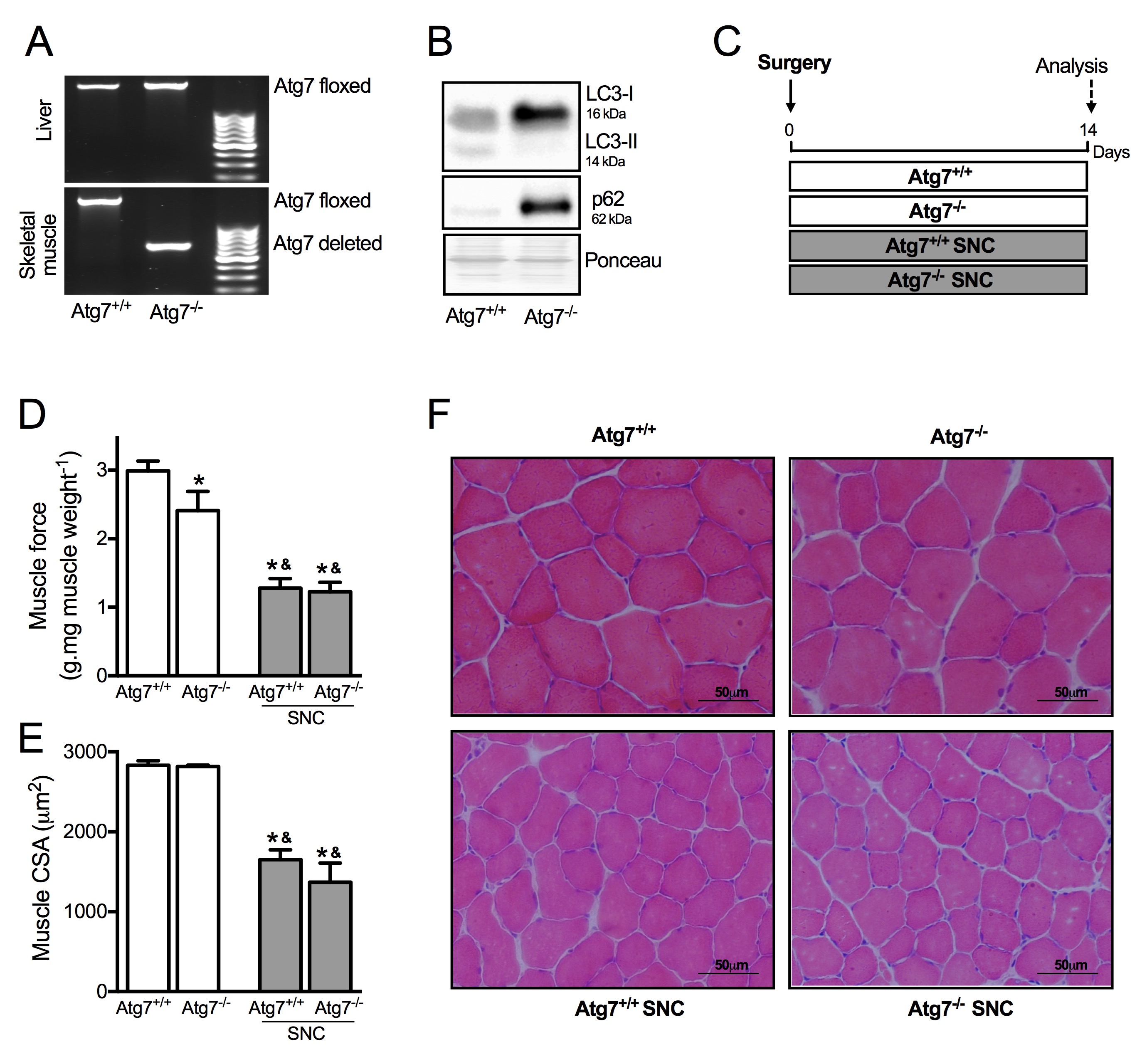


**Supplementary Figure S2.** Genetic autophagy disruption *in vivo* reduces skeletal muscle strength in healthy animals, but not in neurogenic myopathy. Mice bearing *Atg7* Flox alleles (Atg7^f/f^)^1^ were crossed with a muscle-specific, doxycycline inducible *Cre* line (*HSA-rtTA*/*TRE-Cre*)^2^ to generate Atg7^f/f^: *HAS-rtTA/TRE-Cre*. To induce the muscle-specific deletion of Atg7, which is hereafter referred to as Atg7^-/-^, Atg7^f/f^*:HAS-rtTA/TRE-Cre* mice were treated with Doxycycline (1 mg.mL^-1^) for 2 weeks in 5% sucrose-supplemented drinking water, administered to mice in foil-wrapped water bottles, and changed every other day. Atg7^f/f^ mice were also treated with Doxycycline as described above and used as controls (Atg7^+/+^). (**A**) Genomic DNA isolated was subjected to PCR analysis to confirm both genotyping in liver and *Cre*-mediated recombination in skeletal muscle as described previously^3^. (**B**) LC3 and p62 protein representative images of skeletal muscle. Impaired LC3 lipidation and accumulation of p62 protein in Atg7^-/-^ reinforces an efficient *Cre*-mediated recombination of lox-P sites. (**C**) Schematic panel: Atg7^+/+^ and Atg7^-/-^ mice were submitted to sham or SNC groups. At the end of experimental protocol, skeletal muscle morphological and functional analyses were performed. (**D**) *Ex vivo* skeletal muscle function assessed by development of force in response to 150 hertz in EDL muscle. Forces are expressed in grams and normalized by the EDL muscle wet weight. (**E**) Myofiber CSA and (**F**) representative images of tibialis anterior muscle from Atg7^+/+^, Atg7^-/-^, Atg7^+/+^ SNC and Atg7^-/-^ SNC mice. Data are presented as mean ± SEM. Data normality was assessed through Shapiro-Wilk’s test. Two-way ANOVA was applied and statistical significance was considered achieved when the value of P was < 0.05. *, p<0.05 vs. Atg7^+/+^; &, p<0.05 vs. Atg7^-/-^; n=5-8 animals.

**References**

1 Komatsu, M. *et al.* Impairment of starvation-induced and constitutive autophagy in Atg7-deficient mice. *J Cell Biol* **169**, 425-434, doi:10.1083/jcb.200412022 (2005).

2 Rao, P. & Monks, D. A. A tetracycline-inducible and skeletal muscle-specific Cre recombinase transgenic mouse. *Dev Neurobiol* **69**, 401-406, doi:10.1002/dneu.20714 (2009).

3 Masiero, E. *et al.* Autophagy is required to maintain muscle mass. *Cell Metab* **10**, 507-515, doi:10.1016/j.cmet.2009.10.008 (2009).
